# Activities of the oral health teams in primary health care: a time-motion study

**DOI:** 10.1186/s12913-024-11053-5

**Published:** 2024-05-10

**Authors:** Lorrayne Belotti, Sofia Maito, Andrea Liliana Vesga-Varela, Leticia Yamawaka de Almeida, Maira Tamires da Silva, Ana Estela Haddad, Danielle da Costa Palacio, Daiana Bonfim

**Affiliations:** 1 Albert Einstein Center for Studies, Research, and Practices in Primary Health Care and Networks, Hospital Israelita Albert Einstein, Sao Paulo, SP Brazil; 2 School of Dentistry, University of Sao Paulo, Sao Paulo, SP Brazil

**Keywords:** Oral health team, Primary health care, Health human resources, Workload, Time and motion studies

## Abstract

**Background:**

Efficient planning of the oral health workforce in Primary Health Care (PHC) is paramount to ensure equitable community access to services. This requires a meticulous examination of the population’s needs, strategic distribution of oral health professionals, and effective human resource management. In this context, the average time spent on care to meet the needs of users/families/communities is the central variable in healthcare professional workforce planning methods. However, many time measures are solely based on professional judgment or experience.

**Objective:**

Calculate the average time parameters for the activities carried out by the oral health team in primary health care.

**Method:**

This is a descriptive observational study using the time-motion method carried out in five Primary Health Care Units in the city of São Paulo, SP, Brazil. Direct and continuous observation of oral health team members occurred for 40 h spread over five days of a typical work week.

**Results:**

A total of 696.05 h of observation were conducted with 12 Dentists, three Oral Health Assistants, and five Oral Health Technicians. The Dentists’ main activity was consultation with an average duration of 24.39 min, which took up 42.36% of their working time, followed by documentation with 12.15%. Oral Health Assistants spent 31.57% of their time on infection control, while Oral Health Technicians spent 22.37% on documentation.

**Conclusion:**

The study establishes time standards for the activities performed by the dental care team and provides support for the application of workforce planning methods that allow for review and optimization of the work process and public policies.

## Introduction

Despite oral health conditions such as dental caries and periodontal disease being the most prevalent worldwide [1], inequities in dental care coverage and access remain in several countries [2–4]. In Brazil, the integration of Oral Health Teams (OHT) into the Family Health Strategy (FHS) in Primary Health Care (PHC) aimed to improve the population’s access to oral health measures, as the care provided by OHT under the FHS should focus on health promotion, disease prevention, and control and treatment of oral diseases [5].

Other countries, such as the United States, have promoted movements like the “100 Million Mouths Campaign” [6], which aims to create a pilot oral health equity program based on primary care training programs to integrate oral health into their curricula. However, despite the progress and achievements in expanding access to oral health services, there remains a high demand for care from users and an urgent need for changes in work organization.

In this context, the planning of the dental care workforce in PHC is crucial to ensure efficient and accessible dental care for the community [7]. This process involves a careful analysis of the needs of the population of the area, the appropriate distribution of professionals, and the efficient management of human resources [8–10]. A study conducted in Brazil showed that Brazilian regions with the greatest dental needs, such as the North and the Mid-West regions, have the lowest number of dental procedures performed, which draws attention to the need to expand the range and types of procedures offered by the OHT, reinforcing the importance of reducing regional inequalities to enable the population to have access to comprehensive oral health [11]. However, there is a need to make progress in the planning of the dental care workforce in PHC to enable the access of the population to this care.

To this end, there are tools in the literature that can help with this planning. One of these tools is the Workload Indicators for Staffing Need Method (WISN) proposed by the World Health Organization (WHO), which is used in several countries around the world [12], including Brazil [13]. One of the strategies adopted by countries using this method is to determine the proportion of time allocated to each component of the workload [14]. This information is crucial for sizing the health care workforce and for policymakers and managers when making decisions [15]. However, there are few studies that provide objective and empirical measurements of time spent on Dental Care Team interventions/activities in PHC. The aim of this study was therefore to calculate average time parameters for the activities carried out by the dental care team in primary health care.

## Method

This is a descriptive observational study using the time-motion methodology. This continuous observation technique allows for detailed data collection for determining the time professionals spend on each activity during the work day [16].

The study was conducted between March and September 2022 in five Primary Health Care Units (PHCU) in the southern region of the city of São Paulo, SP, Brazil. These units are situated in two administrative districts, Campo Limpo (CL) and Vila Andrade (VA), which together have an estimated population of around 400,000 inhabitants. The area is marked by high social vulnerability, with both districts ranking among the top five with the highest percentage of households in *favelas* in São Paulo in 2021 (VA: 32.7%; CL:21.7%) [17].

The PHCU included in the study operate from Monday to Friday, from 7 a.m. to 7 p.m., and have 14 OHT working in conjunction with the 33 Family Health teams, serving approximately 115,000 individuals within the community. Therefore, each OHT is responsible for an average of 8,200 individuals.

### Population and sample

The study focused on oral health professionals affiliated with the OHT in the five PHCUs under examination. These teams comprise individuals from up to three professional categories: Dentists (professionals with university degrees) Oral Health Technicians, and Oral Health Assistants (professionals with secondary education).

There are currently two types of oral health teams, which differ in team composition: I - Dentist (general practitioner or family medicine specialist) and Oral Health Assistant (OHA) or Oral Health Technicians (OHTs); II - Dentist (general practitioner or family medicine specialist), OHTs, and OHA or other OHTs. The eligibility criteria for this study were therefore: being a professional at an oral health teams, modality I or II [18]. The exclusion criterion was having less than two days of activities monitored.

### Recruitment of participants

Initially, contact was made with the coordination of each PHCU to present the study to the health care professionals. On the scheduled date, the meeting took place in small groups in compliance with the health protocols against COVID-19. After the presentation of the researchers and the main aspects of the study, professionals were invited to participate in the study.

The researchers then sent a QR code that directed the professionals to the Informed Consent Form (ICF). If the professional expressed interest in participating in the study by agreeing to the ICF, they were asked to provide some information about their socio-demographic profile and their work profile. An agreement was then made with the service coordinators and the professionals regarding the period of data collection to observe the activities carried out during the work day.

### Data collection

Data collection was conducted by two researchers holding dentistry degrees, both of whom underwent 160 h of theoretical and practical training specifically for this phase. Direct and continuous observation was conducted of the typical working day of professionals, which consists of 40 h, five days a week. This observation period was important considering the dynamic setting of a PHCU, where oral health professionals engage in a diverse array of tasks, necessitating a comprehensive understanding of their interactions with patients and colleagues.

Additionally, the strategy of continuous observation during five days employed in this study contributes to mitigate the Hawthorne effect. This minimizes the chances of healthcare professionals’ behavior being influenced by their awareness of being observed, thus ensuring a more accurate representation of their daily activities and interactions. Furthermore, this approach facilitates an authentic immersion in the work environment, capturing nuances and details that might otherwise be overlooked by alternative data collection methods [19].

The time-motion technique requires a ratio of one observer per professional, as the researcher accompanies the professional throughout their daily activities. Hence, during the data collection phase, each researcher was responsible for observing one professional per week. The observation, conducted without intervention or interaction, began when the professional arrived at their workplace and ended at the end of their daily shift. Each activity and the time spent performing it were recorded on a portable electronic device used exclusively for the study and stored in the Research Electronic Data Capture (REDCAP), a platform for collecting and managing data [20].

These records were created using the tool proposed by Bonfim et al. (2016) for assessing the workload of healthcare professionals in primary health care [21]. The tool was revised and adapted aiming to detail the activities/interventions carried out by the oral health teams, including a specific item for “oral health consultation”. Therefore, the composition of the tool included 37 health care interventions, related activities, personal activities, waiting time, and absence. The spaces in which the professional carries out their activity (inside and outside the units) were also documented, as well as the activities that were the subject of any interventions during their performance.

### Statistical analysis

The data was exported to an Excel master sheet and validated by the authors for completeness and illogical values. The times for each activity were calculated and adjusted using the following formulas:

1 $$TSI=TO-TI$$

TSI = Total activity time without interruption.

TO = Total observed time of the activity (End time of the activity in minutes - Start time of the activity in minutes).

TI = Time of observed interruptions during the activity period.

2 $$TM= \frac{TSI}{NO}$$

TM = Average activity time.

TSI = Total activity time without interruption.

NO = Number of activity observations.

To calculate TSI, key elements were used: the total observed time of the activity (TO) and the time of observed interruptions (TI). TO is defined as the total duration of the activity, measured in minutes, and is calculated by subtracting the start time of the activity from the end time of the activity. TI represents the total time of interruptions that occurred during the activity period. Therefore, by applying Eq. 1, we can accurately determine how much effective time was devoted to performing a specific activity, excluding the time spent on interruptions.

Eq. 2 allowed calculating the average time of an activity (TM), which was obtained by dividing the total activity time without interruption (TSI) by the number of activity observations (NO). In summary, TM represents how much time, on average, is spent on each execution of the activity, considering the total time devoted to the activity and the number of times that activity was performed.

All results were evaluated using descriptive statistics. The 37 activities observed at the FHS were grouped by position and profession and the average and rate for each activity group were then calculated.

## Results

A total of 696.05 observation hours were undertaken with 20 professionals from the OHT, comprising 12 Dentists (426.37 h), three Oral Health Assistants (103.95 h), and five Oral Health Technicians (165.73 h). Most of the dentists were female (80%), with a mean age of 39.9 years (SD 6.58), about 40% had a Lato-Sensu specialization, 30% had a PhD, and 70% had more than 10 years of experience in PHC. Among the technicians and assistants, the majority were also female (83%), with an average age of 39.7 years (SD 7.6), and 50% had 4 to 6 years of experience in PHC.

Care interventions were carried out most frequently by the three professional categories. Among these interventions, indirect care activities were carried out most frequently, with OHA and OHTs standing out with around 75% and 77%, respectively (Table 1).

**Table 1:** Distribution (absolute and relative frequency) of interventions/activities performed by dental care team professionals

	Dentist		Oral Health Assistant		Oral Health Technician	
	*N*	%	*N*	%	*N*	%
**Interventions**	1702	68.41	530	75.50	642	57.53
Direct care	585	34.37	133	25.09	148	23.05
Indirect care	1117	65.63	397	74.91	494	76.95
**Waiting Time**	88	3.54	27	3.85	59	5.29
**Personal Activity**	566	22.75	88	12.54	275	24.64
**Related Activity**	108	4.34	48	6.84	121	10.84
**Absence**	24	0.96	9	1.28	19	1.70
Total	2600	100.00	702	100.00	1117	100.00

**Direct care**: Care provided directly to the user/family/community; **Indirect care**: care provided away from the user/family/community but for their benefit covering actions focused on management of the unit and interdisciplinary collaboration, for example, documentation and administrative meetings; **Waiting time**: the time during which the professional is available at their workplace to provide care, waiting for the user and/or the professional who is not present at the time of the observation, either because the user is absent and/or late, there is no demand, or the other professional is busy with another activity; **Related Activity**: activities that can be carried out by other professionals from other categories but which are carried out by the health care professional, for example, searching for medical records and exams; **Personal activities**: necessary breaks in the work day to satisfy the physiological and personal communication needs of professionals; **Absence**: when the professional is absent during the work day to carry out activities not related to the USF, such as arriving late and leaving work early.

Among the care interventions carried out by dentists, the average dental consultation lasted 24.39 min and took up 42.36% of the time of this professional group, followed by documentation (12.15%), organization of the work process (6.23%), administrative meetings (5.87%), and the exchange of information about health care and/or health services (5.36%). Infection control (31.57%) and support with exams/procedures (20.92%) were the activities on which OHA spent the most time. Among OHTs, documentation (22.37%), outpatient procedures (8.66%), organization of the work process (6.74%), and exchange of information about health care and/or health services (6.57%) were the main interventions (Table 2). In terms of interruptions in the main activities, 17.49% of dental consultations carried out by dentists were also affected by interruptions. For documentation activities carried out by OHTs, this rate was 22.95%, while for infection control activities carried out by OHA, the interruption rate was 4.97%.

**Table 2:** Absolute frequency, average time in minutes of the activities performed, and the percentage of time spent on the activity according to the professional category

Distribution of activities		Dentist (*n* = 12)			Oral Health Assistant (*n* = 3)			Oral Health Technician (*n* = 5)		
		NO	TM	%	NO	TM	%	NO	TM	%
**Direct Care**	**Interventions**									
	Dental Consultation	444	24.39	42.36	-	-	-	-	-	-
	Dental Consultation – Oral Cancer Campaign	56	2.96	0.65	-	-	-	-	-	-
	Collective procedures	18	10.44	0.74	-	-	-	13	36.23	4.77
	Home Visits	11	20.45	0.88	-	-	-	20	12.55	2.54
	Outpatient Procedures	8	12.37	0.39	6	6.67	0.64	41	20.83	8.66
	Support with exams/procedures	5	12.00	0.23	85	15.35	20.92	26	22.81	6.01
	Promotion of educational activities	1	9.00	0.04	-	-	-	-	-	-
	Community health development	1	2.00	0.01	-	-	-	1	13.00	0.13
	Anthropometric Measurements				-	-	-	1	1.00	0.01
	Advice on the Health Care System	40	2.70	0.42	16	1.75	0.45	33	2.15	0.72
	Care to spontaneous demand	2	10.00	0.08	26	2.08	0.87	14	2.86	0.41
**Indirect Care**	Documentation	337	9.50	12.15	20	6.35	2.04	122	18.09	22.37
	Administrative meetings	25	60.08	5.87	2	67.50	2.16	3	68.67	2.09
	Organization of the work process	216	7.38	6.23	69	6.33	7.01	82	8.11	6.74
	Exchange of information about health care and/or health services	401	3.42	5.36	84	2.39	3.22	217	2.99	6.57
	Scientific research	13	38.46	1.96	1	11.00	0.18	1	3.00	0.03
	Development of care processes and protocols	4	59.75	0.93	-	-	-	-	-	-
	Safety monitoring	7	20.29	0.56	-	-	-	-	-	-
	Multidisciplinary care assessment meetings	4	36.00	0.56	-	-	-	-	-	-
	Infection control	100	1.48	0.58	161	12.23	31.57	50	3.22	1.63
	Mapping and territorialization	1	11.00	0.04	-	-	-	-	-	-
	Health surveillance	7	5.00	0.14	1	37.00	0.59	3	21.67	0.66
	Reference and cross-reference	1	11.00	0.04	-	-	-	-	-	-
	Educational actions for health care professionals (master’s/doctoral courses)	2	103.00	0.81	-	-	-	-	-	-
	Educational actions for health care professionals (permanent training)	4	82.50	1.29	2	18.00	0.58	2	154.50	3.13
	Student support	1	4.00	0.02	-	-	-	-	-	-
	Supply control	3	4.67	0.05	57	5.70	5.21	13	8.46	1.11
**Personal Activity**		566	4.91	10.87	88	3.41	4.81	275	5.50	15.34
**Absent**		24	37.38	3.51	9	94.56	13.64	19	18.32	3.53
**Related Activity**		108	2.55	1.08	48	3.21	2.47	121	4.98	6.11
**Waiting Time**		88	6.28	2.16	27	8.41	3.64	59	12.44	7.44

NO = Number of activity observations; TM = Average activity time

It was observed that part of the technician time was allocated to personal activities, accounting for 15.34% of the total time. In turn, the dentist spent about 10.87% of their time to this type of activity, while the OHA spent about 5% of their time (Table 2).

Initial dental consultations and consultations for procedures/treatment were those with the longest average duration, about 34 and 31 min respectively. Of the total time of consultations, those with spontaneous demand (38.81%), which are also more frequent (*n* = 198), and consultations for procedures/treatments (38.03%) take up the most professional time. A total of 122 complaints were identified during the consultations, of which the main complaint of tooth pain and sensitivity took 44.16% of the time the professionals spent on the consultations. When analyzing consultations by type of procedure performed, the average time was highest for the combination of clinical and surgical procedures (38.33) and lowest for preventive consultations (16.88) (Table 3).

**Table 3:** Absolute frequency, average time in minutes, and percentage of time spent on consultations made by the dentist, according to complaint, type of consultation, and procedure

	*N*	Avg. time	%
**Type of consultation**			
Initial consultation	53	34.06	16.09
Spontaneous demand	198	21.21	38.81
Maintenance consultation	17	14.06	2.43
Consultation for procedure/treatment	126	31.17	38.03
Initial dental consultation for pregnant women	22	8.73	1.81
Shared consultation	12	20.25	2.02
**User’s main complaint**			
Tooth pain and sensitivity	54	20.15	44.16
Fistula, abscess, and gum inflammation	13	19.31	10.18
Fracture	17	25.59	16.76
Prosthesis	8	32.50	10.51
Fall of the restoration	10	20.28	8.47
Third molar	2	15.00	1.25
Others	18	11.72	8.67
**Consultation by type of procedures**			
Preventive* Individual Four-handed	26 10 15	16.88 13.70 19.67	31.54 68.46
Clinical* Individual Four-handed	182 49 125	30.29 28.65 30.89	26.81 73.19
Surgical* Individual Four-handed	60 8 50	30.78 30.75 30.58	14.24 85.76
Preventive + Clinical* Individual Four-handed	30 4 24	29.53 33.00 28.58	16.54 83.46
Clinical + Surgical Individual Four-handed	9 2 7	38.33 43.00 37.00	25.00 75.00
Preventive + Clinical + Surgical Individual Four-handed	5 3 2	23.40 17.67 32.00	45.30 54.70

*Missing in the composition of care variable (Individual or four-handed)

In Table 4, it is possible to observe a detailed breakdown of the intervention that occupied most of the professionals’ time. Among OHA, cleaning equipment and furniture was the most common infection control activity (39.61%). For OHTs, entry in the medical record outside of consultation hours (48.36%) was the most common documentation activity, followed by entry in the production sheet (21.31%).

**Table 4:** Absolute and relative frequency of the other main activities carried out by the professionals

Oral Health Assistant	**Infection control**	**N**	**%**
	Hand washing	15	5.88
	Washing materials for sterilization	18	7.06
	Packing materials for sterilization	21	8.24
	Packaging sterilized material	14	5.49
	Applying the autoclave biological test	8	3.14
	Cleaning equipment and furniture	101	39.61
	Others	78	30.59
	Total	255	100.00
Oral Health Technician	**Documentation**		
	Recording in medical records outside consultation hours	59	48.36
	Administrative	17	13.93
	Typing into monitoring spreadsheets	8	6.56
	Production sheet	26	21.31
	Others	12	9.84
	Total	122	100.00
Dentist	**Documentation**		
	Administrative	18	5.50
	Typing into monitoring spreadsheets	8	2.46
	Production sheet	144	44.04
	Recording in medical records outside consultation hours	143	42.81
	Recording in medical records after home visit	1	0.31
	Others	17	4.89
	Total	327	100.00
	**Exchange of information about health care and/or health services**		
	Case discussion outside consultation hours	95	23.69
	Between consultations (in-service discussion)	30	7.48
	Exchange of information about work processes	212	52.87
	Others	64	15.96
	Total	401	100.00
	**Organization of the work process**		
	Indicator analysis	1	0.36
	Preparation of shift schedules	1	0.36
	Organization of the appointment book	67	23.93
	Reply to work emails	23	8.21
	Reply to work WhatsApp	46	16.43
	Others	142	50.71
	Total	280	100.00

For dentists, besides consultation, documentation, information exchange, and organization of the work process are among the five most important interventions on which they spend the most time. In terms of documentation, those related to recording the production sheet (44.04%) and recording in the medical record outside of consultation hours (42.81%) stood out. Regarding interventions related to the exchange of information about health care, there was emphasis on exchanging information about work processes (52.87%), while in interventions related to the organization of the work process, the organization of the appointment book showed a frequency of around 24% (Table 4).

Most of the monitored activities took place within the PHCU facilities, with a notable prevalence in the tasks performed by OHA (99.29%), followed by the dentist (97.22%), and the OHTs (92.20%). Inside the PCHU, the dentist’s office was the main location where these activities were carried out, with rates of 79.78%, 79.07%, and 66.95% for dentists, OHTs, and OHA respectively. In addition, around 11.95% of the activities performed by OHA took place in the sterilization and rinsing room. For dentists and OHTs, the corridor was the second most frequently used space for carrying out activities at 3.20% and 5.06%, respectively. Additionally, the meeting room, as well as the Community Health Agents (CHA) and Family Health Support Center (FHSC) rooms are rarely used by the dental care team.

## Discussion

The results indicate that the dental care team in PHC spends more time during their work day on interventions related to direct and indirect care. The number of indirect care interventions was significant in all three professional categories. However, the majority of the dentist’s working time was spent on direct care, particularly dental consultations. Oral health assistants and technicians spent more time on indirect care activities, particularly infection control and documentation, respectively.

An average duration of around 24 min was recorded for the consultation intervention, with the longest average time recorded for initial dental consultations. The values found are below the recommendations for dental consultations in PHC in the city of São Paulo, which vary between 40 and 60 min, depending on the type of procedure to be performed, the complexity of the case, and the dental care team involved in the consultation [22]. However, note that on average more than eight thousand people are assigned to each of the teams involved in this study. This large number of users, combined with the high pressure of care, may pose a challenge in meeting the recommendations for average dental consultation time.

A study with dentists working in primary care in the United Kingdom’s National Health System (NHS) found that the average total time spent on preventive procedures such as oral hygiene advice, polishing, and fluoride varnish application was around 18 min. Surgical procedures such as surgical preparation and tooth extraction, on the other hand, took longer, totaling 25.9 min [23]. Although the study analyzed the procedures individually, the average times are consistent compared to the present study.

According to dental professionals’ assessment of the factors that affect performance times, besides the complexity of the procedures, various elements influence the fluctuations in average times. These include the type of appointment scheduling system, reception management, the characteristics of the instruments, the presence of a clear treatment plan, and the quality of the dental materials. Furthermore, it is important to emphasize individual factors such as skills and experience besides leadership and team work skills [24].

It was observed that spontaneous demand consultations and consultations related to complaints of toothache and tooth sensitivity were the ones that took up the most time during the consultation intervention. A high spontaneous demand reflects the need to expand access to oral health care in PHC, in order to ensure the attribute of first contact and strengthen the bond between users and oral health teams. In the context of PHC in Brazil, the challenge of oral health care lies in balancing the high suppressed demand requiring immediate attention with the need to ensure continuity of care, alongside preventive and promotional oral health activities [25, 26]. The results of this study enable a discussion on workforce planning to ensure comprehensive oral health care delivery.

The documentation intervention had a significant impact on working hours, particularly for dentists and oral health technicians. The average time spent on documentation in this study was found to be higher than the averages reported by dental care teams in the UK, which ranged from 5.8 to 7.1 min [23]. This discrepancy can be attributed to the practice of physical record-keeping, where professionals write down the information on paper and then enter it into the system. However, a study conducted in the US found that even in scenarios where the record is fully electronic, approximately 54% of direct interaction time with the patient during primary care medical consultations was spent recording in the electronic medical record [27].

In the Brazilian context, health care professionals often use different information systems in their daily work to record and retrieve relevant data about users. A study conducted in Brazil from 2013 to 2018 found that there were 31 national health care information systems in use in the PHC, 15 of which did not have unified interfaces with the Electronic Citizen’s Medical Record [28]. The need to fill multiple systems that are fragmented among themselves leads to rework and can eat up time that could be spent on direct user care.

Moreover, despite the oral health technician devoting a significant portion of their work to interventions in indirect care, it is crucial to recognize their potential in providing direct user assistance. These professionals can play a key role in performing preventive procedures such as fluoride application, cleaning and polishing of teeth, removal of plaque, etc [29]. Research suggests that greater use of the dental care team’s skills mix, including delegation and substitution of simple routine tasks to the oral health technician, can be an efficient way to increase staff capacity and access to dental care and give dentists more time for complex tasks [24, 30]. However, in PHC, in line with the findings of this study, it is observed that in addition to the limited integration of the oral health technician in services, professionals often perform dental assistant functions, with a greater involvement in indirect care activities [31, 32].

Among the most common indirect care interventions of the dentist, information exchange about health care stood out. This intervention plays a key role in improving communication, strengthening working relationships between team professionals, and promoting collaborative practices. In addition, interprofessional collaboration can improve the quality of care, as it enables a more comprehensive and integrated understanding of users’ needs, facilitates the coordination of services and helps to avoid duplication of efforts, improves the efficiency of the health care system, and ensures a more user-centered approach [33].

Despite the infrequency, home visits by the dentist and the oral health assistant stand out. This practice, while more common in the routine of other FHS professionals, has the potential to enhance access to dental care, especially for individuals with mobility challenges, such as the elderly, people with disabilities, or those in situations of social vulnerability [34, 35]. Furthermore, home visits allow for a deeper understanding of users’ socio-economic conditions, oral hygiene habits, and cultural aspects, thereby providing more personalized care, tailored to their specific needs [36].

However, organizing home care through the oral health teams remains a challenge and home visits in the PHC are still in their infancy, primarily due to a lack of prioritization and the perception that the dentist’s work is focused on the clinical procedures in the office. The home environment is characterized by its de-institutionalized, complex, and multifaceted nature, which requires oral health practice that goes beyond the exclusively clinical and procedural approach traditionally used in dentistry [37].

Although a high frequency of personal activities was observed, especially among dentists and oral health technicians, the time spent on these activities was relatively short, about five minutes, resulting in a lower use of the total time of the work day. Similar to the results of this study, a multicenter survey of general practitioners in Portugal found that about 12% of the working time of these professionals was spent on personal activities [15]. The inclusion of time spent on these activities is essential for realistic workforce projections, as it reflects real-world dynamics and contributes to a more comprehensive understanding of the demands and challenges faced by professionals in PHC.

The identified parameters on the professional practice of the dental care team represent important information for decision makers, especially for leaders in different governance areas. In this sense, given the constantly evolving oral health issues and technological advances, it is essential to adopt a dynamic approach and management strategies to meet the population’s needs [7]. This encompasses leveraging technology to streamline and automate administrative tasks, establishing streamlined work protocols that optimize the practice, and implementing collaborative practice models that foster teamwork and information sharing among healthcare professionals. This data can be effectively utilized for strategic planning and assessing staffing levels, as well as aiding decisions concerning the redistribution of resources among healthcare units. Furthermore, the time shares identified for each category not only underpin planning, but also provide a solid basis for organizing the work process between the different professionals, resulting in a more efficient approach to the delivery of dental care [14].

The data collection took place during the COVID-19 pandemic, which may have had an impact on the activities and distribution of the team’s working hours. The pandemic had a significant negative impact on the supply of dental treatments in the Brazilian Unified Health System, leading to a severe reduction in the number of procedures performed [38, 39]. Furthermore, the implementation of additional safety protocols against COVID-19, such as patient screening, rigorous equipment disinfection procedures, and more frequent and lengthy cleaning intervals, likely required additional time and resources, leading to adjustments in the team’s workflow and priorities [40].

Additionally, it is important to consider that activities carried out quickly by professionals, such as hand hygiene, may not be accurately captured during data collection. However, direct observation by external observers provided more accurate and reliable data compared to subjective methods such as self-reporting. Furthermore, it contributes to studies of the time-motion technique in the health care area, as in developing countries, with information systems that are still fragile, capturing parameters is still necessary based on investments in research that use robust techniques, such as the one used in the study, to propose real-world parameters. Therefore, also note that this study provides an unprecedented overview of the current interventions/activities developed by oral health professionals, as well as the average times spent in different situations, complaints, types of procedures, and number of professionals involved. This information is essential for properly sizing and optimizing the workforce planning.

## Data Availability

The data underlying this study contain potentially sensitive information, and sharing them openly could compromise the privacy and confidentiality of the study participants. In accordance with ethical guidelines and to protect participant confidentiality, the raw data cannot be made publicly available. However, interested researchers may request access to a de-identified and aggregated dataset by contacting the authors. Requests will be considered on a case-by-case basis, and approval will be contingent upon ensuring the continued protection of participant privacy.
